# Parental Satisfaction with Caregiving for Children with Developmental Disabilities: Development of a New Assessment Tool

**DOI:** 10.3390/children5120166

**Published:** 2018-12-11

**Authors:** Sayyed Ali Samadi, Ghasem Abdollahi-Boghrabadi, Roy McConkey

**Affiliations:** 1 Institute of Nursing and Health Research, Ulster University, N. Ireland and the Medical Sciences Division, Academic Center for Educational, Cultural and Research (ACECR)—Shahid Beheshti University, Tehran 1985717443, Iran; 2 Department of Psychology, Payame Noor University, Tehran 19569, Iran; psy-abdollahi@pnu.ac.ir; 3 Institute of Nursing and Health Research, Ulster University, Newtownabbey BT37 0QB, Northern Ireland, UK; r.mcconkey@ulster.ac.uk

**Keywords:** parental satisfaction, caregiving, developmental disabilities, autism, intellectual disabilities, ADHD

## Abstract

Parents of children with developmental disabilities face many challenges. Those who are less satisfied with the care-giving that their children receive may require extra support. This paper reports the development of a Parental Satisfaction with Caring for a child with Developmental Disability Index (PSCDDI), with items derived from literature reviews and pilot testing in Iran. The index was field tested with 256 parents caring for children with a diagnosis of intellectual disability, autism, or attention deficit hyperactivity disorder (ADHD), and who were attending centres in four locations across Iran. A factor analysis of responses to the self-completion questionnaire identified two main factors, i.e., personal satisfaction and satisfaction with the child, with six items for each factor. The two factors had good internal consistency and reasonable test-retest reliability. Binary logistic regressions identified significant predictors of personal satisfaction that supported the validity of the scale.: Clinicians and service providers could use this scale as part of their assessment strategy to identify parents who may require additional support, especially those with teenage female children, and those with a diagnosis of autism or behavior problems.

## 1. Introduction

Bringing up a child with a developmental disability (DD) is a task with both positive and negative impacts on parents and other family members [1,2]. Their satisfaction with caregiving for children with DD is an important factor in the overall healthcare of children. Support services provided for children need to boost parental satisfaction, and should attune their assistance to parental needs and expectations [3].

Considerable research has been conducted examining the adverse effects of caring for a child with a developmental disability such as autism spectrum disorders (ASD) [4] on mothers at a higher risk of being negatively impacted [5,6,7]. A possible reason for such adverse effects could be that mothers play a more crucial role in caring, rearing, and the education of children with disabilities than do fathers or other family members [8].

There is growing interest in studying the impact on parents of having children with DD, especially in low and middle-income countries, where support services are not well developed. Most of the studies in both western and non-western cultures have been focused on the impact on mothers, with very little research on fathers [9,10]. Considering fathers as well as mothers in all types of services for the parents of children with DD, such as understanding their ideas and perspective, would be important when considering or addressing their particular needs.

Although the challenging aspects of raising a child with a developmental disability are well documented [11], as Taunt & Hastings [12] pointed out, there are anecdotal data available from parents and other family members to indicate that these impacts could also include positive dimensions that might be critical in building parental resilience. This dual impact has been studied in some cultures such as India, in which both positive and negative impacts were reported. These impacts appear to be closely related to cultural values that varied from viewing the child with a disability as a gift and blessing from God, to attributing the disability to one’s own or the child’s karma [13]. 

Parents’ personal attitude is a key issue in nurturing the development of children with disabilities. Mothers and fathers may adopt different or opposite attitudes towards their children and the impacts they have on their lives. Savage and Bailey [14], in a review of the available literature on the impact of caregiving for a child with disabilities, showed that a lower level of satisfaction with caregiving is common among parents of children with developmental disabilities. However, they also reported that other studies discovered positive impacts on parents of caregiving for a child with disabilities. Parents reported acting in a way that brought happiness to the care recipient, maintaining their dignity, and maximizing the potential of the care recipient [15].

Cultural values play a significant role in how parents perceive developmental disabilities, which in turn influences parental responses to caregiving. The stigma of having a child with a developmental disability is a major challenge facing parents in some cultures [16]. For example, Indian mothers of children with developmental disabilities often blame themselves for their child’s conditions, and they considered illness during pregnancy and poor pre- and postnatal care as the cause of their child’s disability [17]. Shobana and Saravanan [18], in a comparison between three groups of mothers of children with developmental disabilities, reported that parents who exhibited negative attitudes towards their children’s disabilities were found to believe that the situation is due to immorality or the consequence of sins and karma committed in the past. These findings were reconfirmed by Mourya, et al., [19] in India. Similar findings have been reported from western societies such as Sweden, in which parents of young children with disabilities viewed their child’s condition negatively, and by adopting this attitude, negatively affected their emotional well-being [20].

Research evidence also clearly indicates the extra demands that caring places on carers. Kuhn and Carter [21] investigated the associations between maternal self-efficacy and parenting cognitions—that is, the thoughts, attitudes, and beliefs that parents have about their parenting of children with ASD. Their efficacy as care-givers was influenced by their upbringing, parenting experiences, and interactions with particular children. In sum, parental satisfaction with their caring role might be considered as an important factor in the overall quality of life of families who have a child with developmental disabilities.

Presently, there is a dearth of studies on parental satisfaction with the caring for a child with developmental disabilities in Middle Eastern countries. The present study sought to devise a scale that clinicians and service providers could use to identify families who are struggling with their caregiving role. Such a scale would help researchers to gain a better understanding of the factors associated with caregiving satisfaction, and to identify the contributing factors that make caregiving a positive experience for parents.

## 2. Materials and Methods

### 2.1. Methodology

The Parental Satisfaction with Caring for a child with Developmental Disability Index (PSCDDI) was designed for use by clinical and service providers to identify areas where parents need support with parenting skills, and to assess the effect of family-based interventions on parents’ satisfaction with their role as caregivers. The 14 items were initially based on a review undertaken by Hastings & Taunt [22]. The index was piloted in the first author’s PhD thesis “The impact on Iranian parents who have children with an Autism Spectrum Disorder (ASD)” (2010) [23] and in a study with parents of children with different types of developmental disabilities and ordinary developing children [24]. The items were acceptable to parents and understood by them.

A self-completion questionnaire was devised that listed the 14 items. Parents were requested to rate each item using a five-point Likert scale, ranging from “strongly agree” (with score 1), “agree” (score 2), “not sure” (score 3), “disagree” (score 4) and “strongly disagree” (score 5). In addition, demographic details of the parents and child were requested.

### 2.2. Data Collection 

Overall, 21 centres were approached (12 special centres in Tehran province city of Tehran, 5 centres in Isfahan province city of Kashan and 4 centres in Kurdistan province city of Sanandaj). Children with intellectual disabilities (ID), attention deficit hyperactivity disorders (ADHD) and ASD attended the centres, and all had a confirmed developmental disability registered with the Iranian Social Welfare Organization (ISWO). Ethics Filter Committee of the School of Nursing (University of Ulster), 20 October 2007.

Two trained data collectors then visited the centres and met with parents who had been asked to attend. The study was explained to parents and questionnaires were distributed to those who wished to take part. Out of 276 disseminated questionnaires, 20 parents did not respond (7.2%) giving a sample of 256 parents. Their demographic characteristics and those of the children are given in Table 1 and Table 2.

Four months later, 70 parents were asked to complete the scale for a second time in order to assess its test-retest reliability. This sample was drawn from the same locations and included children with different impairments.

### 2.3. Data Analysis

The information from the questionnaires was analysed using Statistical Package for the Social Sciences (SPSS) version 24 (IBM, Armonk, NY, USA). The questionnaire data was entered and analysed by the first author and cross checked by the third author. A factor analysis was undertaken of the 14 items, and summary scores were calculated for the two factors that had been identified. Internal reliability was assessed using Cronbach’s α and Pearson Product Moment correlations for test-retest reliability. Univariate analyses were used to identify possible predictors of parental satisfaction which were then confirmed using binary logistic regressions.

## 3. Results

### 3.1. Item Analysis

Table 3 summarises the percentage of parents who had “strongly agreed” with each item subdivided into those with younger and older children. Although parents’ responses tended to be skewed towards agreement, the items did discriminate among the parents with the percentage of respondents selecting “strongly agree” varying across the subsamples in Table 3 from a low of 18.8% to a high of 87.5%.

A factor analysis of parental ratings of the 14 items using Varimax rotation with Kaiser normalization identified two main factors which, together, accounted for over 60% of the variance. The first factor (34% of variance) had six items relating to personal satisfaction with factor loadings >0.700. The second factor (26% of variance) comprised six items relating to satisfaction with the child with factor loadings close to or >0.600. Table 3 summarises the items and their factor loadings.

In addition, there were two items that had low loadings on both factors (<0.600): “My child has strengthened our family and/or marriage” and “Having a child has led me to develop new skills” These items were omitted from further analyses.

### 3.2. Summary Scores

A summary score could be calculated for each factor by adding the ratings to each item that was given by the parents. For each factor, the summary scores could range from 6 to 30. A higher score was indicative of less satisfaction. The mean score on the personal factor was 11.3 (standard deviation (SD) 4.6, range 6–24) and for the child factor the mean score was 9.6 (SD 3.5, range 6–22). The difference between the two sets of scores was statistically, different with parents obtaining greater satisfaction from their child than personally (paired *t*-tests *t* = 7.75, *p* < 0.001).

The Pearson product moment correlation between the personal satisfaction scores and the child satisfaction scores was *r* = 0.636 (*p* < 0.001). Although significant, the shared variance was only around 40%.

The internal consistency across the six items in each factor was assessed using Cronbach’s α. For personal satisfaction, it was 0.889. and for child satisfaction, it was 0.837. These results suggest a high degree of consistency across the items in each factor.

Test-retest reliability was assessed by correlating a subsample of the parents’ scores (*n* = 70) on the satisfaction measures calculated on their first and second administration of the scale. Across all 12 items, the Pearson product moment correlation was *r* = 0.824: (*p* < 0.001). However, the correlations were lower for the two factors, although still significant: personal satisfaction *r* = 0.741: child satisfaction *r* = 0.704. There were no statistically-significant differences in the mean scores at the two time points on satisfaction scores.

### 3.3. Relationships between Satisfaction Scores and Predictor Variables

The sensitivity of the satisfaction measures was tested by examining the relationship between the satisfaction measures and possible predictor variables such as parent and child gender, child’s age, type of developmental disability, and the child behavior problems. Binary logistic regressions were used in order to control for the inter-correlations among the predictor variables. Parents were grouped into those with low and high satisfaction scores based on the median of the total group. 

The regression for the personal satisfaction scores was significant (*χ*^2^ = 66.83; degrees of freedom (df) = 8; *p* < 0.001; Nagelkerke = 0.307). The parents who had less personal satisfaction were those with female children, children aged 12–17 years, and those with a diagnosis of autism and who exhibited behavior problems. 

The regression for the child satisfaction scores was also significant (*χ*^2^ = 38.83: df = 8: *p* < 0.001: Nagelkerke = 0.189). However, the only significant predictor was the child’s gender: with parents of female children being less satisfied (*p* < 0.01).

These analyses provide some evidence for the validity of the scale, as the findings concur with those in previous literature. (Further details of the regression analyses are available from the first author).

## 4. Discussion

This study reports the development of a scale that can be used to assess parental satisfaction with caregiving when a child has a developmental disability. Moreover, it extends existing knowledge about caregiving satisfaction by identifying two aspects associated with it: personal satisfaction from caregiving, and satisfaction with having the child.

The 12-item index was easily administered, and proved acceptable to both mothers and fathers across different locations in Iran who had children with different developmental problems. The index had strong internal reliabilities, but somewhat weaker test-retest reliabilities. The latter may have resulted from the overly long gap, i.e., four months, during which time, real changes may have occurred in parental caregiving. 

The index identified some significant relationships between parents’ personal satisfaction and child characteristics. In particular, parents reported less satisfaction in caregiving for female children, those aged 12–17 years, or when the child had autism or showed behavior problems. These results echo findings from previous research, and they help to confirm the validity of the scale. Interestingly, parents’ rating of satisfaction was not affected by these characteristics except for gender. Hence the scale could be used by clinicians to identify parents at particular risk of low carer satisfaction. Moreover, the index might be used to monitor changes as a result of interventions provided to the child and family such as parent education course, counselling, or therapy programs.

The lower satisfaction reported by Iranian parents of female children is worthy of closer attention. The reasons may be rooted in Iranian cultural beliefs, which indicate that compared to boys, girls are more fragile, with a submissive status, and need more protection and help [25]. Any type of disabilities may, therefore, increase the need for caring and supervision of girls, and place extra pressures on parents. Sabih and Sajid [26] reported similar findings in their study of Pakistani parents of children with ASD. In their study, the parents of girls with ASD had higher stress scores which caused them to be less satisfied with caring compared to parents who had a boy with autism.

Also, religious beliefs may influence parental perceptions of dealing with a child’s disability. There are some interpretations of Islamic ideas that consider disabilities as a punishment from God, resulting from a committed sin [27] or as the conventional consequences of divine will [28], which is similar to notions found in other eastern Asian cultures [29].

Further research could usefully focus on fathers. Although their mean satisfaction ratings in this sample were not significantly different from those of mothers, other studies report different impacts on mothers and fathers [26,30]. However, mother-father pairs may report differences that clinicians need to take into account.

Parenting satisfaction with their caregiving might act as a significant predictor of parenting stress [2]. Just as with parental levels of stress, their level of satisfaction with caring may also change over time. Hauser-Cram et al. [31] (2001) reported that following families of children with DD from early stages of diagnosis through to 10 years of age, parental stress drastically increases, and by the time to child is 10 years old, four times as many parents were reporting stress in the clinical range compared to parents in the typically developing sample. This is also reported in other Middle Eastern cultures [26]. Hence, the index could be used longitudinally with families as part of regular reviews.

Previous research suggests that having positive perceptions about the children’s condition helps parents to cope with the challenges and difficulties associated with bringing up a child with DD. For example, Iranian parents reported increased self-confidence and a sense of satisfaction after educational sessions in which they learnt about caring for a child with developmental disabilities [7,32]. Likewise Probst [33] also found improved parent and child interactions after parents attended educational sessions, which can be considered as an indicator of parental satisfaction with their caregiving role.

There are some limitations to this study. The data were collected in only three provinces across Iran. Given that the sample was drawn from multiple centres across these provinces, we are confident that it represents these places sufficiently, but it may or may not be representative of the broader population of parents of children with different types of DDs across the country or the region. Further study of a wider geographic area, alongside an evaluation of the demographic characteristics of the respondents, is planned. This will also include further measures of parental health, stress, and coping, in order to better understand the inter-relationships among the various parental indicators, and further define the validity of the index.

It is also possible that additional items could enhance the sensitivity of the index. A qualitative study is currently underway to examine the experiences of Iranian parents in terms of what gives them satisfaction in their care-giving and the difficulties they encounter. From these new items, factors might be identified that are especially suited to Iranian and Middle Eastern Cultures.

Similarly, an increasing number of carers are looking after adult persons with developmental disabilities. To date, few studies in non-western countries have examined their satisfaction with the prolonged period of caregiving they have to undertake.

In conclusion, clinicians could use this scale as part of their assessment strategy to identify parents who may require additional support and to understand the effect of interventions at the parental level on satisfaction with caregiving for a child with DD. This study demonstrates the value of using family measures to better understand and support parents with a child with a DD. Furthermore, this research may assist policymakers and professionals in Iran and other countries with similar culture to improve the service provisions and to improve awareness of the needs for this group of parents.

## Figures and Tables

**Table 1:** Parents’ demographic details.

Gender	City in Which Parents Live	Age
Male 164 (64%) Female 92 (36%)	Tehran (city of Tehran) 83 (32%) Kurdistan (Sanandaj) 45 (18%) Isfahan (Kashan) 128 (50%)	Under 37 years old 140 (55%) Above 37 years old 116 (45%)

**Table 2:** Children demographic information.

Gender	Order of Birth	Type of DD	Age (years)	Having Behavioural Problems Based on Parental Reports	Additional Diagnosis
Male 208 (81%) Female 48 (19%)	1st 135 (53%) 2nd 75 (29%) 3rd 28 (11%) 4th 6 (2.3%) 5th 8 (3.1%) 6th 4 (1.6%)	ID = 80 (31.2%) ASD = 68 (26.6%) ADHD = 108 (42.2%)	3 to 12 = 128 (50%) 13 and above = 128 (50%)	Yes 132 (52%) No 122 (48%)	Yes 56 (22%) No 200 (78%)

DD: developmental disorders; ID: intellectual disability; ASD: autism spectrum disorders; ADHD: attention deficit hyperactivity disorders.

**Table 3:** The item loadings on the two factors and the frequency and percentage of the items which parents rated as ‘strongly agree’ based on the child’s age.

Loadings on Personal Factor	Loadings on Child Factor	Item	*N* (%) Child Aged 4–12 Years (*N* = 128)	*N* (%) Child Aged 13–17 Years (*N* = 128)	*N* (%) All Children (*N* = 256)
0.831	0.204	I make the most of each day; live life at a slower pace	66 (51.6%)	35 (27.3%)	101 (39.5%)
0.770	0.307	My personal strength and confidence has increased	59 (46.1%)	36 (28.1%)	95 (37.1%)
0.751	0.227	My perspective on life has changed—I am more aware of what is important in life	77 (60.2%)	43 (33.6%)	120 (46.9%)
0.744	0.115	My spirituality or trust in God has increased	78 (60.9%)	49 (38.3%)	127 (49.6%)
0.743	0.316	I have become a better person—less selfish, more tolerant, compassionate	80 (62.5%)	44 (35.5%)	124 (48.4%)
0.730	0.209	My social and community networks have expanded	63 (49.2%)	24 (18.8%)	87 (34.0%)
0.112	0.807	I get a sense of accomplishment in having done one’s best for the child	105 (82.0%)	74 (57.8%)	179 (70.0%)
0.129	0.789	I get pleasure in providing care for my child	112 (87.5%)	69 (53.9%)	181 (70.7%)
0.298	0.683	My child is a source of joy and happiness in my life	82 (64.1%)	47 (36.7%)	129 (50.4%)
0.311	0.674	My child provides me with a challenge and an opportunity to learn and develop	70 (54.7%)	41 (32.0%)	111 (43%)
0.278	0.602	My child gives me love and affection	91 (71.1%)	52 (40.6%)	143 (55.9%)
0.570	0.587	My child gives me a new or increased sense of purpose in life	82 (64.1%)	44 (34.9%)	126 (49.2%)
0.599	0.501	Having a child has led me to develop new skills	71 (55.5%)	30 (23.4%)	101 (39.5%)
0.537	0.422	My child has strengthened our family and/or marriage	66 (51.6%)	32 (25.0%)	98 (38.3%)

Note: The items are re-ordered by factor loadings. In the revised questionnaire, the items should be randomized and the two shaded items should be omitted.
